# A comparative planning study of step-and-shoot IMRT versus helical tomotherapy for whole-pelvis irradiation in cervical cancer

**DOI:** 10.1093/jrr/rrv004

**Published:** 2015-02-26

**Authors:** Imjai Chitapanarux, Ekkasit Tharavichitkul, Wannapa Nobnop, Somsak Wanwilairat, Roy Vongtama, Patrinee Traisathit

**Affiliations:** 1 Division of Therapeutic Radiology and Oncology, Department of Radiology, Faculty of Medicine, Chiang Mai University, Chiang Mai, Thailand; 2 St Teresa Comprehensive Cancer Center, Stockton, CA, USA; 3 Department of Statistics, Faculty of Science, Chiang Mai University, Chiang Mai, Thailand

**Keywords:** IMRT, step-and-shoot, tomotherapy, cervical cancer

## Abstract

The aim of this study was to compare the dosimetric parameters of whole-pelvis radiotherapy (WPRT) for cervical cancer between step-and-shoot IMRT (SaS-IMRT) and Helical Tomotherapy™ (HT). Retrospective analysis was performed on 20 cervical cancer patients who received WPRT in our center between January 2011 and January 2014. SaS-IMRT and HT treatment plans were generated for each patient. The dosimetric values for target coverage and organ-at-risk (OAR) sparing were compared according to the criteria of the International Commission on Radiation Units and Measurements 83 (ICRU 83) guidelines. Differences in beam-on time (BOT) were also compared. All the PTV dosimetric parameters (D5%, D50% and D95%) for the HT plan were (statistically significantly) of better quality than those for the SaS-IMRT plan (*P*-value < 0.001 in all respects). HT was also significantly more accurate than SaS-IMRT with respect to the D98% and Dmean of the CTV (*P*-values of 0.008 and <0.001, respectively). The median Conformity Index (CI) did not differ between the two plans (*P*-value = 0.057). However, the Uniformity Index for HT was significantly better than that for SaS-IMRT (*P*-value < 0.001). The median of D50% for the bladder, rectum and small bowel were significantly lower in HT planning than SaS-IMRT (*P*-value < 0.001). For D2%, we found that HT provided better sparing to the rectum and bladder (*P*-value < 0.001). However, the median of D2% for the small bowel was comparable for both plans. The median of Dmax of the head of the left femur was significantly lower in the HT plan, but this did not apply for the head of the right femur. BOT for HT was significantly shorter than for SaS-IMRT (*P*-value < 0.001). HT provided highly accurate plans, with more homogeneous PTV coverage and superior sparing of OARs than SaS-IMRT. In addition, HT enabled a shorter delivery time than SaS-IMRT.

## INTRODUCTION

Cervical cancer is the second most common cancer among women in Thailand; 9999 new cases were diagnosed in 2008 [1]. A significant survival benefit has been demonstrated for the combined approach of concurrent chemoradiotherapy (CCRT) [2–4]. The radiation therapy component consists of external beam irradiation to the primary tumor and regional lymph nodes, followed by a brachytherapy boost to the gross tumor in the cervix. However, acute Grade 3 or 4 hematological and gastrointestinal toxicities were found to be significantly higher in the CCRT group than in the radiotherapy (RT) alone group. Tan *et al.* [5] also demonstrated increased late toxicity after CCRT for locally advanced cervical cancer.

To combat this increase in toxicity, whole-pelvic intensity-modulated radiotherapy (WP-IMRT) has been applied to gynecologic malignancies with excellent planning target volume (PTV) coverage and this has been associated with less acute gastrointestinal sequelae than conventional whole-pelvic radiotherapy (WPRT), as reported by Mundt et al. [6]. It has been demonstrated that, with the same target coverage, IMRT is superior to conventional techniques in normal tissue sparing for the treatment of cervical cancer, with lower gastrointestinal, genitourinary, and bone marrow toxicity [7–11]. Whole-pelvic step-and-shoot (SaS) IMRT for cervical cancer patients has been used in our center since 2007. A newer technique for delivering IMRT, helical tomotherapy™ (HT) (using a continuously rotating beam), can provide highly conformal dose distributions and simultaneous critical organ-sparing ability [12, 13]. It is being studied for use in gynecologic malignancies and thus far has provided promising results with respect to reliable set-up accuracy and a reduction in planning margin [14]. In our center, SaS-IMRT started in the year 2007, and HT (TomoTherapy Hi-Art with treatment planning system (TPS) Hi- Art version 4.2.1) commenced in 2012. We use both techniques in our practice. This is the first dosimetric comparison study of the two techniques in WPRT for locally advanced cervical cancer in our country.

## MATERIALS AND METHODS

A total of 20 cervical cancer patients who received whole-pelvis irradiation with the SaS-IMRT technique at the Division of Therapeutic Radiology and Oncology, Chiang Mai University, between January 2011 and January 2014 were retrospectively identified.

For IMRT planning, a computed tomography (CT) simulation was performed. Vac-Loc was used to immobilize patients in position. To prepare the bladder, patients were advised to urinate 20 min before scanning and to drink 200 ml of water after voiding. Patients were then set up in CT. For rectal preparation, a laxative was administered in case of rectal dilatation. This protocol was used during irradiation to maintain bladder and rectal volume. With the patients in supine treatment position and legs relaxed on the table, the pelvic region from the L1–L2 interspace and covering the whole vagina was scanned without intravenous contrast to obtain appropriate images. The CT slice thickness was 5 mm (no interslice gap). In our center, we have only one 12-year-old CT-simulator unit, which has a single slice thickness for IMRT planning (Toshiba: Asteion). To preserve the machine, a 5-mm slice thickness was selected (for a large volume site such as for pelvic irradiation).

After imaging was completed, image data was sent for contouring (Oncentra Masterplan®, Nucletron, an Elekta company, Elekta AB, Stockholm, Sweden) and planning (KonRad treatment planning software®, Siemens, Concord, CA, USA).

The RTOG/JCOG recommendations were used in combination as a guide for contouring the clinical target volume (CTV) [15–18]. The CTV was composed of the cervix, uterus, adnexae, upper half of the vagina, and pelvic lymph nodes (common iliac lymph nodes (LNs), external iliac LNs, internal iliac LNs, obturator LNs and presacral LNs). The PTV was defined as the CTV plus a 0.7-cm margin (for the pelvic lymph nodes) and a 1–1.5-cm margin (for the primary cervical tumor). The bladder, rectum, sigmoid colon, small bowel and heads of femurs were contoured as organs at risk (OARs). For the PTV, the dose of 1.8–2 Gy per fraction at five fractions per week was prescribed (i.e. a total dose of 45–46 Gy). The D95 (dose to 95% of the volume) of the PTV was calculated. Additional to the doses to 5% of the bladder, rectum and small bowel, the maximal doses to the heads of the femurs were also evaluated. As described earlier, patients were advised to prepare the bladder and rectum for treatment as for the CT simulation. An electronic portal imaging device (EPID) was used weekly to evaluate and make corrections to positioning.

An SaS-IMRT plan was generated for each patient using KonRad, Siemens TPS, with seven coplanar step-and-shoot beams at fixed gantry angles G1 to G7. The G1 to G7 for most cases were 0, 40, 80, 125, 235, 280 and 320 degrees, respectively. For only six of the patients, G4 and G5 were 5-degrees modified to 120 and 240 degrees, respectively. All patients received SaS-IMRT whole-pelvis irradiation with our Siemens Primus.

A HT plan was then created using the Tomotherapy treatment-planning system (HiArt, Tomotherapy, Tomotherapy Inc., Madison, WI) for every patient (by the same medical physicist who planned the SaS-IMRT). The optimization parameters of dose constraint and overlap priority were given the same consideration in both the SaS-IMRT plan and the HT plan.

We used the same contouring as for SaS-IMRT for plan generation. Both plans were required to use identical planning objectives with respect to optimal PTV coverage and OAR sparing. Plans were designed using a field width of 5 cm, a pitch of 0.287 and modulation factor of 2.2 to 2.8. The mean setting and actual modulation factor were 2.235 and 2.032, respectively.

Dosimetric parameters were analyzed for each technique. D50%, D95% and D5% of the PTV were selected. The priorities were prescribed as follows: PTV, bladder, rectum, sigmoid colon, small bowel, and head of femur. For organs-at-risk, D50% and D2% to the small bowel, rectum and bladder were considered. The Dmax of the bilateral heads of the femurs were also analyzed. To assess the uniformity of dose distribution in the PTV, we calculated a uniformity index (UI) from the formula UI = D5/D95, where D5 and D95 were the minimum doses delivered to 5% and 95% of the PTVs. The ideal value is 1, and it increases as the plan becomes less homogeneous. We also calculated the quality of coverage, with a conformity index (CI) that was defined as the ratio between the target volume and the target volume that was covered by the reference isodose. A CI = 1 corresponds to ideal conformation. A CI > 1 indicates that the irradiated volume is greater than the target volume. A CI < 1 indicates that the target volume is only partially irradiated.

A direct comparison of the dosimetric parameters between SaS-IMRT and HT was also performed (using the Wilcoxon's matched pairs test) to determine if there was a significant difference for any of the parameters examined. Differences were considered statistically significant at *P* < 0.05. Additionally, beam-on timtes (BOTs) (defined as the time from first beam on until the last beam was turned off) of the two delivery systems were also evaluated and compared. The study was approved by our Institutional Review Board. All analyses were performed using SPSS version 17.

## RESULTS

### Patient characteristics

A total of 20 women were included, with a median age of 55.5 years (IQR, 52–63 years). All patients belonged to FIGO Stage IIB and IIIB and received concurrent chemoradiotherapy with weekly cisplatin at 40 mg/m^2^. All of the patients were treated with WP SaS-IMRT followed by brachytherapy or external beam radiotherapy according to the tumor status after WPRT. Baseline patient characteristics were shown in Table 1. The dosimetric data for SaS-IMRT and HT are shown in Tables 2 and 3, respectively.

**Table 1.** Patient characteristics

No.	Age	Stage	Dose prescription
1	51	IIB	45 Gy/25 Fx
2	51	IIB	45 Gy/25 Fx
3	62	IIB	45 Gy/25 Fx
4	63	IIB	45 Gy/25 Fx
5	53	IIB	45 Gy/25 Fx
6	46	IIIB	45 Gy/25 Fx
7	52	IIB	45 Gy/25 Fx
8	57	IIB	45 Gy/25 Fx
9	52	IIB	45 Gy/25 Fx
10	54	IIB	45 Gy/25 Fx
11	52	IIB	45 Gy/25 Fx
12	53	IIIB	45 Gy/25 Fx
13	61	IIB	45 Gy/25 Fx
14	57	IIB	45 Gy/25 Fx
15	63	IIB	45 Gy/25 Fx
16	73	IIB	46 Gy/23 Fx
17	70	IIB	46 Gy /23 Fx
18	67	IIIB	46 Gy /23 Fx
19	53	IIB	46 Gy /23 Fx
20	72	IIB	46 Gy /23 Fx

**Table 2.** The data for SaS-IMRT

No.	Bladder		Rectum		Head of femur		Bowel		CTV				PTV				
	D50 (Gy)	D2 (Gy)	D50 (Gy)	D2 (Gy)	Dmax Lt (Gy)	Dmax Rt (Gy)	D50 (Gy)	D2 (Gy)	Dmean (Gy)	D98 (Gy)	CI	UI	D5 (Gy)	D50 (Gy)	D95 (Gy)	CI	UI
1	41.2	48.5	37.2	49.2	46.9	45.1	28.5	46.2	48.0	45.2	0.981	1.103	50.1	47.5	43.4	0.925	1.155
2	44.9	47.5	41.3	47.5	45.2	45.4	30.5	44.8	47.8	45.4	0.986	1.084	49.6	47.7	44.3	0.941	1.119
3	41.4	48.2	38.3	49.0	47.4	43.6	21.9	37.6	47.8	45.1	0.981	1.107	50.1	47.5	43.9	0.938	1.141
4	42.9	48.9	37.0	48.8	44.8	41.9	28.9	41.5	48.5	45.9	0.989	1.093	50.3	48.1	44.8	0.944	1.124
5	40.5	48.0	36.6	48.6	38.3	39.0	26.3	40.0	48.1	45.8	0.986	1.094	50.1	47.7	44.5	0.942	1.128
6	41.1	48.8	43.5	50.3	42.3	40.9	31.2	46.5	48.7	46.2	0.994	1.084	50.5	48.3	44.3	0.941	1.139
7	41.3	50.3	35.9	52.0	47.4	47.7	28.6	41.8	49.8	47.0	0.999	1.095	51.9	49.6	44.9	0.949	1.156
8	40.2	51.0	40.6	50.8	48.0	48.0	24.9	41.8	49.5	46.2	0.998	1.108	51.8	49.1	45.0	0.951	1.152
9	48.0	50.8	34.6	52.0	45.9	45.3	27.4	41.0	49.7	47.2	0.999	1.096	51.8	49.1	44.9	0.950	1.153
10	40.5	49.5	37.9	48.8	47.3	47.1	25.2	39.6	49.2	46.7	0.996	1.091	51.2	48.7	45.1	0.952	1.136
11	37.5	49.9	40.4	50.0	48.8	46.5	28.2	41.8	49.7	46.8	0.994	1.101	51.9	49.4	44.9	0.949	1.155
12	38.1	48.9	36.2	46.4	45.5	43.7	26.2	40.8	49.9	47.0	0.998	1.100	52.0	49.4	45.0	0.950	1.156
13	40.4	50.8	36.7	50.9	44.2	41.1	29.7	40.5	49.3	46.2	0.992	1.113	51.6	48.7	44.6	0.942	1.157
14	37.1	49.7	40.2	49.8	44.3	43.8	28.0	39.2	49.3	46.5	0.996	1.092	51.2	48.8	45.0	0.952	1.137
15	38.5	50.4	35.9	50.8	46.1	43.9	23.5	38.9	49.9	47.0	1.000	1.095	52.0	49.6	45.4	0.960	1.146
16	41.9	52.8	41.1	50.7	44.4	43.0	29.6	49.0	50.7	48.0	0.994	1.100	52.6	50.1	45.7	0.945	1.152
17	42.4	51.1	44.2	52.2	42.0	41.8	31.1	49.2	49.0	46.3	0.982	1.111	51.4	48.7	45.0	0.944	1.142
18	39.7	50.1	42.0	49.2	38.8	41.0	22.1	47.5	48.9	45.8	0.976	1.099	50.9	48.6	45.0	0.942	1.131
19	44.4	49.9	41.3	48.8	42.3	42.6	22.9	48.6	49.0	45.2	0.967	1.113	51.3	48.7	44.9	0.938	1.144
20	42.2	50.3	37.9	48.8	44.1	42.0	29.4	49.2	48.9	46.5	0.982	1.093	50.9	58.5	45.6	0.947	1.117

**Table 3.** The data for HT

No.	Bladder		Rectum		Head of femur		Bowel		CTV					PTV			
	D50 (Gy)	D2 (Gy)	D50 (Gy)	D2 (Gy)	Dmax Lt (Gy)	Dmax Rt (Gy)	D50 (Gy)	D2 (Gy)	Dmean (Gy)	D98 (Gy)	CI	UI	D5 (Gy)	D50 (Gy)	D95 (Gy)	CI	UI
1	34.3	45.3	33.9	46.5	41.1	41.5	24.1	45.1	46.1	45.3	0.995	1.028	46.7	46.0	44.8	0.942	1.042
2	43.9	46.0	39.6	46.7	44.7	45.8	27.2	45.3	46.3	45.4	0.992	1.028	46.8	46.2	44.9	0.949	1.042
3	36.6	46.8	39.2	46.7	44.3	43.5	21.0	40.4	46.6	45.4	0.987	1.029	47.3	46.5	44.8	0.942	1.056
4	38.7	47.1	32.7	46.1	43.0	42.8	26.6	42.3	46.5	45.5	0.990	1.029	47.0	46.4	44.9	0.950	1.047
5	38.2	46.8	34.0	45.7	43.8	42.0	25.9	41.4	47.2	46.0	0.994	1.034	47.8	47.1	45.0	0.950	1.062
6	38.8	46.2	41.4	47.3	43.7	43.4	23.7	45.4	46.4	45.3	0.989	1.032	47.0	46.3	44.6	0.940	1.054
7	41.3	46.8	33.8	46.7	45.1	44.4	26.3	41.1	46.4	45.7	0.999	1.026	47.0	46.3	45.1	0.956	1.042
8	37.1	46.4	38.2	46.4	44.7	44.7	24.1	40.3	46.2	45.2	0.992	1.028	46.8	46.1	45.0	0.950	1.040
9	44.8	46.6	33.7	46.9	44.7	43.9	26.0	40.6	46.4	45.6	0.992	1.027	47.0	46.3	45.1	0.951	1.042
10	36.7	46.5	31.5	46.6	43.7	43.5	22.7	41.8	46.4	45.6	0.999	1.025	46.9	46.2	44.9	0.946	1.045
11	38.3	47.0	38.6	47.6	44.3	44.2	26.6	40.9	46.7	45.9	0.997	1.027	47.2	46.6	44.9	0.946	1.051
12	36.1	46.6	35.9	46.2	42.0	42.6	23.7	44.3	46.3	45.4	0.994	1.027	46.8	46.1	44.9	0.950	1.042
13	38.7	46.8	35.3	46.3	44.0	43.1	27.2	41.9	46.5	45.7	0.999	1.026	47.0	46.3	44.8	0.947	1.049
14	38.4	46.3	36.2	46.6	43.6	42.8	26.0	41.1	46.3	45.4	0.996	1.027	46.9	46.2	44.9	0.949	1.045
15	38.1	46.6	34.1	46.2	43.0	43.3	21.0	40.7	46.0	45.2	0.995	1.025	46.6	46.0	44.9	0.944	1.038
16	35.5	47.7	38.8	49	42.0	44.1	28.6	47.5	47.0	46.3	0.991	1.035	48.0	47.2	45.8	0.948	1.048
17	30.1	47.9	40.6	48.1	39.9	40.2	30.1	47.5	47.4	46.7	1.000	1.024	48.1	47.5	46.0	0.951	1.046
18	33.3	47.1	35.4	47.5	42.0	40.8	18.6	46.1	47.0	46.4	0.997	1.025	47.8	46.9	45.7	0.942	1.046
19	42.7	47.8	37.2	47.8	41.0	41.7	20.3	46.7	46.9	46.0	0.985	1.032	47.8	46.9	45.9	0.949	1.041
20	31.7	47.4	30.8	45.4	44.7	43.6	24.3	47.3	47.0	46.2	1.000	1.027	47.6	46.9	45.9	0.946	1.037

**Fig. 1.**
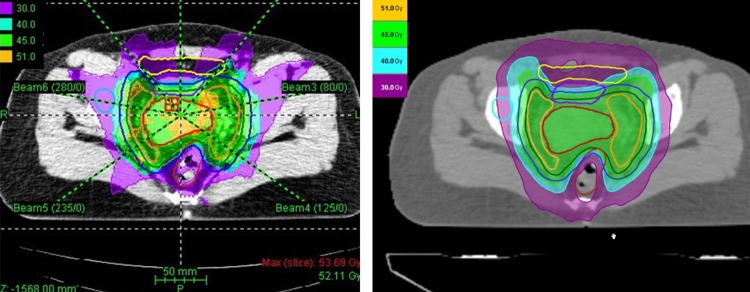
Dose distribution for SAS-IMRT (left) and HT (right) plan.

### Target coverage

The dosimetric parameters for the HT and IMRT plans were analyzed (Table 4). Dose distribution in all SaS-IMRT and HT plans for all 20 patients satisfied clinical requirements. The dose distributions for SaS-IMRT and HT planned for one patient are shown in Fig 1. HT planning demonstrated a Dmean and D98% closer to the prescription dose than SaS-IMRT planning, with a *P*-value of < 0.001 and 0.008, respectively. All the PTV dosimetric parameters (D5%, D50% and D95%) of the HT plan were of significantly better quality than for the IMRT plan. The dose conformity and the CI did not differ between the plans, but the target dose UI of the HT plan was significantly better than that of the SaS-IMRT plan.

**Table 4.** Wilcoxon matched pairs test between the two treatment plans

Structure	DVH criteria	Tomotherapy median (IQR)	SaS-IMRT median (IQR)	*P*-value
Age			55.50 (52.00–63.00)	
Bladder	D50	38.15 (35.80–38.75)	41.15 (39.95–42.30)	<0.001
	D2	46.80 (46.45–47.10)	49.90 (48.85–50.60)	<0.001
Rectum	D50	35.65 (33.85–38.70)	38.10 (36.65–41.20)	<0.001
	D2	46.65 (46.25–47.40)	49.50 (48.80–50.80)	<0.001
Small bowel	D50	25.10 (23.20–26.60)	28.10 (25.05–29.50)	<0.001
	D2	42.10 (41.00–45.75)	41.80 (40.25–47.00)	0.823
Head of femur	Dmax Left	43.70 (42.00–44.60)	45.00 (42.75–47.20)	0.021
	Dmax Right	43.35 (42.15–44.05)	43.65 (41.82–45.38)	0.225
CTV	Dmean	46.45 (46.30–46.95)	49.10 (48.60–49.70)	<0.001
	D98	45.60 (45.40–46.00)	46.25 (45.80–46.90)	0.008
	CI	0.99 (0.99–1.00)	0.99 (0.98–1.00)	0.057
	UI	1.03 (1.03–1.03)	1.10 (1.09–1.10)	<0.001
PTV	D5	47.0 (46.8–47.75)	51.25 (50.35–51.88)	<0.001
	D50	46.30 (46.20–46.90)	48.70 (48.15–49.40)	<0.001
	D95	44.90 (44.90–45.55)	44.90 (44.53–45.00)	<0.001
	CI	0.95 (9.94–0.95)	0.94 (0.94–9.95)	0.093
	UI	1.05 (1.04–1.05)	1.14 (1.13–1.15)	<0.001
Beam-on time		230.6 (217.4–240.1)	900.0 (840.0–904.0	<0.001

### OARs

HT showed better OAR dose sparing than SaS-IMRT. For the radiation dose to the bladder and rectum, the D50% and D2% of HT planning were both significantly less than for the SaS-IMRT plan (*P*-value < 0.001). Regarding the dose to the small bowel, D50% for the HT plan was also statistically less than for the SaS-IMRT plan (*P*-value < 0.001). However, we did not find a statistically significant difference in D2% for the small bowel when comparing the two techniques. The Dmax of the head of the left femur was (statistically significantly) lower in the HT plan, whereas there was no difference for the head of the right femur between the two techniques. The dose statistics of the OARs are also listed in Table 4.

### Beam-on time

The median BOT was significantly shorter in HT delivery (230.6 s) than in SaS-IMRT delivery (900.0 s), with a *P*-value of < 0.001.

## DISCUSSION

For the treatment of cervical cancer, WPRT has been used for more than five decades. The conventional two- or four-field box techniques were applied to the target, which was composed of the primary site and the pelvic LNs. Although this simple technique yielded good treatment results and acceptable toxicity, the OARs still received significant dosages, even with good blocking. With new techniques of imaging (CT or MRI) and planning, the possibility of keeping the target dose high and reducing the dose to the OARs has become a reality. After the first publication concerning the use of IMRT in gynecological cancers by Portelance *et al.* [7], the use of high-technology radiation therapy soon became more prevalent. Most studies have reported the benefits of IMRT in reducing the dose to OARs in radical and postoperative settings. [6, 7, 19–22].

Moreover, the supported study from Cancer Care Ontario reported comparative clinical outcomes for three dimension conformal radiotherapy (3DCRT) and IMRT. This report supported the use of IMRT in the treatment of gynecologic cancers in order to reduce the toxicity of the treatment. However, this group warned that clinicians should be aware of the uncertainties involved and be judicious in the use of gynecologic IMRT with the primary goal of reducing toxicity [23]. The development of intensity-modulated arc therapy (IMAT) was the next area of advancement in technique. The new variation of dynamic-IMRT allowed 360° treatment. HT, volumetric-modulated arc therapy (VMAT™) and RAPID arc™ are all IMAT technologies that were developed to improve treatment quality compared with conventional IMRT. Indeed, a promising trend has been observed, demonstrating IMAT provides better dose distributions and CIs with shorter BOTs when compared with conventional IMRT [24–26].

This study provides a dosimetric analysis of SaS-IMRT and HT to assess optimal treatment planning for pelvic irradiation in locally advanced cervical cancer. We performed the comparative planning studies by independently optimizing planning in the same patient. Our results indicate that HT had better conformal CTV and PTV coverage and sparing of OARs than did SaS-IMRT, as shown in Table 4. HT demonstrated a 3 Gy lower median dose at D2% of the bladder and rectum with statistically significant differences. HT did demonstrate a 1 Gy higher dose Dmean of the small bowel, which was concerning, although it was not significant statistically. HT was of clear benefit in lowering the cumulative bladder and rectal dose when compared with brachytherapy. All the PTV dosimetric parameters (D5%, D50% and D95%) for the HT plan were of significantly better quality than for the IMRT plan. Although the D98 of the CTV had a 1-Gy difference (45.6 versus 46.27; *P* = 0.008), the UI of HT was significantly closer to 1 than SaS-IMRT (*P* < 0.001) with the same CI (0.99). This demonstrates a lower overdose region for treatment by HT. After much discussion between radiation oncologists and medical physicists in our center, we have just this year changed the plan evaluation from D95 to D50 of the PTV, as per ICRU 83. So, D95 of the PTV was the main evaluation for the target, but we evaluated additional parameters as per ICRU 83 [27] recommendations. Although the median dose at D95 of the PTV had statistical significance, all plans achieved the target dose to D95 of the PTV, and HT planning showed better UI and less data deviation than SaS-IMRT. Moreover, our findings suggest that using HT will have a favorable impact on BOT.

Another limitation in using pelvic SaS-IMRT for cervical cancer is organ motion, which is not evaluated prior to treatment delivery. This would be another advantage of using HT: using megavoltage CT before each treatment session, thus allowing the plan to have smaller margins as well as lower radiation exposure to nearby OARs because of improved set-up accuracy and reproducibility.

## CONCLUSION

When treating cervical cancer with IMRT, HT provided a distinct advantage compared with SaS-IMRT with respect to target coverage and OARs. Moreover, it had shorter delivery treatment time.

## CONFLICT OF INTEREST

The authors have declared that there are no conflicts of interest.

## FUNDING

Funding by Faculty of Medicine, Chiang Mai University. Funding to pay the Open Access publication charges for this article was provided by Faculty of Medicine, Chiang Mai University.
